# Complex Metabolomic Changes in a Combined Defect of Glycosylation and Oxidative Phosphorylation in a Patient with Pathogenic Variants in *PGM1* and *NDUFA13*

**DOI:** 10.3390/cells14090638

**Published:** 2025-04-25

**Authors:** Silvia Radenkovic, Isabelle Adant, Matthew J. Bird, Johannes V. Swinnen, David Cassiman, Tamas Kozicz, Sarah C. Gruenert, Bart Ghesquière, Eva Morava

**Affiliations:** 1 Laboratory of Applied Mass Spectrometry, Department of Molecular and Cellular Medicine, KU Leuven, 3000 Leuven, Belgium; s.radenkovic@umcutrecht.nl (S.R.);; 2 Laboratory of Hepatology, Department of Chronic Diseases, Metabolism and Ageing, KU Leuven, 3000 Leuven, Belgium; 3 Metabolomics Core Facility, Center for Cancer Biology, VIB, 3000 Leuven, Belgium; 4 Department of Clinical Genomics, Mayo Clinic, Rochester, MN 55901, USA; 5 Section Metabolic Diagnostics, Department of Genetics, UMC Utrecht, 3584 Utrecht, The Netherlands; 6 Clinical Department of Laboratory Medicine, University Hospitals Leuven, 3000 Leuven, Belgium; 7 Laboratory of Lipid Metabolism and Cancer, Leuven Cancer Institute and Leuven Institute for Single Cell Omics, Department of Oncology, KU Leuven, 3000 Leuven, Belgium; 8 Metabolic Center, University Hospitals Leuven, 3000 Leuven, Belgium; 9 Department of Genetics and Genomics Sciences, Icahn School of Medicine at Mount Sinai, New York, NY 10029, USA; 10 Department of General Pediatrics, Adolescent Medicine and Neonatology, Medical Center, Faculty of Medicine, University of Freiburg, 79106 Freiburg, Germany

**Keywords:** congenital disorder of glycosylation, Leigh syndrome, inborn errors of metabolism, PGM1, NDUFA13, metabolomics

## Abstract

Inherited metabolic disorders (IMDs) are genetic disorders that occur in as many as 1:2500 births worldwide. Nevertheless, they are quite rare individually and even more rare is the co-occurrence of two IMDs in one individual. To better understand the metabolic cross-talk between glycosylation changes and deficient energy metabolism, and its potential effect on outcomes, we evaluated patient fibroblasts with likely pathogenic variants in *PGM1* and pathogenic variants in *NDUFA13* derived from a patient who passed away at 16 years of age. The patient presented with characteristic of PGM1-CDG including bifid uvula, muscle involvement, abnormal glycosylation in blood, and elevated liver transaminases. In addition, hearing loss, seizures, elevated plasma and CSF lactate and a Leigh-like MRI brain pattern were present, which are commonly associated with Leigh syndrome. PGM1-CDG has been reported in about 70 individuals, while *NDUFA13* deficiency has so far only been reported in 13 patients. As abundant energy is essential for glycosylation, and both *PGM1* and *NDUFA13* are linked to energy metabolism, we sought to better understand the underlying biochemical cause of the patient’s clinical presentation. To do so, we performed extensive investigations including tracer metabolomics, lipidomics and enzymatic studies on the patient’s fibroblasts. We found a profound depletion of UDP-hexoses, consistent with PGM1-CDG. Complex I enzyme activity and mitochondrial function were also impaired, corroborating complex I deficiency and Leigh syndrome. Further, lipidomics analysis showed similarities with both PGM1-CDG and OXPHOS-deficient patients. Based on our results, the patient was diagnosed with both PGM1-CDG and Leigh syndrome. In summary, we present the first case of combined CDG and Leigh syndrome, caused by (likely) pathogenic variants in *PGM1* and *NDUFA13*, and underline the importance of considering the synergistic effects of multiple disease-causing variants in patients with complex clinical presentation, leading to the patient’s early demise.

## 1. Introduction

Inherited metabolic diseases (IMDs) are a large group of genetically inherited disorders affecting metabolism. The worldwide prevalence of IMD, based on various reports, is estimated to be approximately 1:2500 [1]. According to the international classification of inherited metabolic disorders (ICIMD), there are 1450 disorders classified as IMDs, which can be hierarchically divided in 24 categories and 124 groups based on the pathway they affect [2]. As the number of the IMDs keeps growing, their classification becomes more and more challenging. While IMDs typically impair the activity of the affected enzyme and pathways closely associated with it, it is not well understood how individual IMDs affect the whole metabolism. The complex interplay between different biochemical pathways is also reflected in overlapping clinical presentations of disorders not classified under the same umbrella. Combined, the above-mentioned factors complicate the IMD diagnosis, and the expertise of a multi-disciplinary team is often necessary to find the correct diagnosis for affected individuals. In some cases, the co-occurrence of two genetic disorders can further complicate the diagnosis of a patient. Although extremely rare, the co-occurrence of two IMDs has increasingly been reported over the years, especially in families with consanguinity [3,4,5].

Analogously, here we report on a patient whose complex clinical presentation prompted a suspicion of IMD. The results of genetic testing further complicated the diagnosis as (likely) pathogenic variants in both *PGM1* and *NDUFA13* genes were found (Table 1).

**Figure 1:**
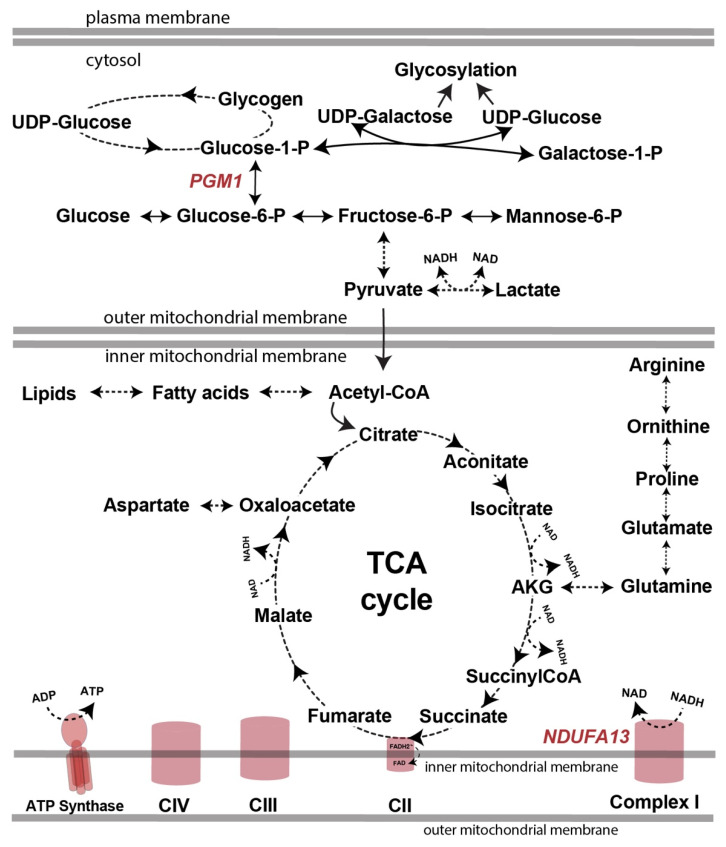
Simplified scheme of glycolysis, glycogen metabolism, TCA cycle and respiratory chain complexes, and the positioning of PGM1 and NDUFA13 within them. Abbreviations: AKG—alpha-ketoglutarate; C—complex; FAD—flavine adenine dinucleotide; NAD—nicotinamide adenine dinucleotide; P—phosphate; TCA—tricarboxylic acid cycle; and UDP—uridine diphosphate.

Pathogenic variants in the *NDUFA13* gene, which codes for one of the subunits of respiratory chain complex I (CI) (Figure 1), have so far been reported in 11 families and 13 patients [6,7,8] and are associated with Leigh syndrome, a progressive encephalopathy defined by symmetrical lesions of the basal ganglia, which are easily spotted with brain imaging (Leigh-like MRI brain pattern).

Leigh syndrome is a severe neurological disorder, which can be caused by pathogenic variants in more than 75 genes (both nuclear and mitochondrial) involved in oxidative phosphorylation (OXPHOS) and OXPHOS assembly, stability and activity. The average frequency of Leigh syndrome is 1:40,000 births and the most common pathogenic variants causing Leigh syndrome are found in genes associated with mitochondrial CI [9,10,11]. Leigh syndrome has a variable onset and can manifest as early as the neonatal period. The classical presentation of Leigh syndrome usually manifests before 2 years of age and includes hypotonia, epilepsy, respiratory distress, developmental delay, ataxia and lactic acidosis, among others. The clinical presentation of late-onset Leigh syndrome can be variable and can mimic other disorders such as multiple sclerosis (MS). Behavior abnormalities and/or psychiatric findings, as well as intellectual decline, movement disorder, headaches and memory loss, can occur. Apart from characteristic central nervous system (CNS) involvement, heart, liver, gastrointestinal, hematological involvement and dysmorphic features can be present [9] (Table 1).

Clinical presentation of so far reported NDUF13A deficient patients [6,7,8] includes slow disease progression, global developmental delay, speech delay or absence of speech, failure to thrive, oculomotor abnormalities, spasticity/hypertonia, axial hypotonia, truncal hypotonia, seizures, movement abnormalities, progressive cerebellar atrophy with hypersignal of the cerebellum and dentate nucleus, metabolic acidosis, elevated lactate on MRS, regression, gastrointestinal involvement and heart involvement, among others (Table 1). The disease in the majority of the patients (11/13) had infantile onset (<12 months) and the mean age of the reported patients was 7.8 years (±5.4 years). One patient died during infancy [8]. To date, no curative treatment for *NDUFA13* deficiency has been proposed. Finally, apart from decreased complex I deficiency [6,7,8], the biochemical consequences of NDUFA13 deficiency have not been thoroughly assessed.

Pathogenic variants in the *PGM1* gene are associated with phosphoglucomutase-1 (PGM1)-congenital disorders of glycosylation (CDG) [12,13]. PGM1-CDG is caused by a deficiency of PGM1 enzyme, which affects multiple biochemical pathways including glycolysis, glycosylation, glycogen synthesis and glycogenolysis (Figure 1). Due to its involvement in glycogen metabolism, PGM1 has initially been classified as a glycogen storage disorder (GSD-XIII) [14]. Glycosylation abnormalities in patients with variants in *PGM1* were reported later, and the disorder was classified as a CDG [12]. Up to date, more than 70 individuals diagnosed with PGM1-CDG have been reported [12,15,16,17,18]. The most common clinical presenting signs include abnormal glycosylation in blood, rhabdomyolysis, increased transaminase concentrations (ALT, AST), hypoglycemia, coagulopathy, abnormal thyroid and sex hormones, dysmorphic features (bifid uvula, Pierre-Robin Sequence, cleft palate), exercise intolerance, muscle weakness, cognitive impairment, increased creatine kinase (CK) and heart involvement, among others [16]. In contrast to other CDGs, individuals with PGM1-CDG usually have normal brain MRI findings and intellectual disability is less common [19] (Table 1). The oldest reported PGM1-CDG patient was 53 years of age at the time of the publication [20]. Sudden death due to cardiac complications was reported in several pediatric patients [16,21,22], but the majority of the patients survive into adulthood [16].

Biochemically, PGM1 deficiency leads to abnormal glycosylation due to the depletion of UDP-glucose and UDP-galactose pools (UDP-hexose) [18]. These metabolites are directly involved in glycosylation by acting as sugar donors of glucose and galactose to growing glycan chains in endoplasmic reticulum (ER) and Golgi apparatus (GA), respectively. PGM1 deficiency, therefore, affects both ER and GA glycosylation, resulting in a distinct mixed CDG glycosylation profile, which is easily detected by carbohydrate-deficient transferrin (CDT) testing in blood [12,23]. The abnormal glycosylation in PGM1-CDG is treatable by oral D-galactose supplementation (Gal), which increases UDP-hexose pools and usually results in the improvement of glycosylation in blood, normalization of CK levels, improvement of coagulation, liver transaminases and hypoglycemia [12,15,18,24,25,26].

Our patient presented with bifid uvula, muscle involvement, abnormal glycosylation in blood, elevated liver transaminases, elevated plasma and CSF lactate and Leigh-like MRI brain pattern (Table 1), indicative of both disorders. To establish the diagnosis, we performed extensive investigations including tracer metabolomics, lipidomics and enzymatic studies on the patient’s fibroblasts. Our results show a complex biochemical phenotype supported by the deficiencies in both PGM1 and NDUFA13. Unfortunately, our patient passed away at 16 years of age.

## 2. Methods

### 2.1. Ethics

Informed research consent was collected from the patient/guardians. Analysis of the fibroblasts at the University Hospitals Leuven were conducted in accordance with ethics application number S60206 (‘Retrospective metabolic analysis of archived fibroblasts’). 

### 2.2. Clinical Biochemical Investigations

Standard biochemical testing of patient plasma/CSF/urine and the enzymatic studies on the muscle biopsy were performed as a part of routine clinical testing. Carbohydrate-deficient transferrin (CDT) testing was conducted as a part of the clinical work-up in the Metabolic Laboratory of the Children’s Hospital of the University of Freiburg, according to standardized protocols.

### 2.3. Genetic Testing

Whole exome sequencing was performed as part of the routine clinical work-up.

### 2.4. Cell Culture

Fibroblasts were collected by skin-punch biopsy. Demographic and molecular characteristics of patients whose fibroblasts were harvested are given in Supplementary Table S1. Healthy control, OXPHOS complex I (CI)-deficient, PGM1-CDG and PGM1/NDUFA13 fibroblasts at less than passage 15 were maintained in low glucose 5.5 mM (physiological) glucose (Glc), 2 mM glutamine (Gln), 10% Fetal Bovine Serum (FBS) DMEM medium (ThermoFisher, catalogue number 31885023) at 37 °C, 5% CO_2_. Routine mycoplasma testing was performed.

### 2.5. Oxygraphy and Enzymology

Cells for both enzymology and oxygraphy were cultured to a density of 50–80% in low glucose 5.5 mM (physiological) glucose (Glc), 2 mM glutamine (Gln), 10% Fetal Bovine Serum (FBS) DMEM medium (ThermoFisher, catalogue number 31885023). Next, they were harvested by trypsinization, washed twice in PBS and either re-suspended to 20 million cells/mL for oxygraphy, or snap-frozen in a dry ice ethanol slurry for enzymology. Oxygraphy and enzymology were performed as previously described [27]. Enzyme activity was normalized to cell count or citrate synthase activity. The data were represented as relative abundances (fold change, FC) compared to healthy controls.

### 2.6. ^13^C_6_-Glucose and Galactose Tracer Studies

Tracer metabolomics in the patient and control (CTR) fibroblasts were performed as previously described [18,28]. Briefly, 15.000 fibroblasts per well were seeded in 6-well plates in 5.5 mM Glc, 2 mM Gln 10% FBS DMEM medium. After 24 h, the medium was changed to either (A) 5.5 mM ^13^C_6_-Glc, 2 mM ^12^C_6_-Gal; (B) 5.5 mM ^12^C_6_-Glc, 2 mM ^13^C_6_-Gal; (C) 5.5 mM ^12^C_6_-Glc, 2 mM ^12^C_6_-Gal; (D) 5.5 mM ^13^C_6_-Glc; or (E) 5.5 mM ^12^C_6_-Glc, 2 mM ^12^C_5_-Gln, 10% FBS DMEM medium. One medium change was performed 48 h later.

All conditions were performed in duplicates. Before harvesting, cells were washed with ice cold 0.9% saline buffer and kept on ice. Cells were scraped in 250 µL of extraction buffer (80% MetOH, 2 µM d27 myristic acid) and transferred to 1.5 mL Eppendorf. Next, the cells were pelleted by centrifugation at 15,000 rpm, 4 °C, 20 min. The supernatant was transferred to a MS vial and subjected to liquid chromatography/mass-spectrometry (LC/MS), while the pellet was used for the protein quantification (BCA Pierce kit, ThermoFisher catalogue number 23225).

### 2.7. Relative Quantification of Metabolites by LC/MS

LC/MS was performed as previously described [18,28]. Briefly, 10 μL of sample was separated on an ion-pairing liquid-chromatography column, and the metabolites were resolved on a Q Exactive Hybrid Quadrupole-Orbitrap Mass Spectrometer in negative ion mode (ESI settings: 50 sheet gas flow rate, 10 auxiliary gas flow rate, 4 kV spray voltage, S-lens RF 60 level, 350 °C capillary temperature). A full scan (140,000 resolution at 200 *m*/*z*, AGC at 3 × 10^6^, 512 ms ion fill time, and 70–1050 *m*/*z* scan range) was applied. Run time per sample was 40 min. The data analysis was performed by using Xcalibur (Thermo Fisher) or El-Maven (Elucidata, v0.11.0) software. The in-house metabolite library was used to annotate metabolites based on their *m*/*z* ratio and retention time (RT). The signal intensity was normalized to IS and protein content. Z-scores were calculated for metabolite abundances and fractional contribution of ^13^C_6_-glucose or ^13^C_6_-galactose. Z-score values using 95% intervals were used (z-score values >1.96 or <−1.96 were considered “significant” or comparable to *p* < 0.05 in a two-tailed test).

### 2.8. Lipidomics

Briefly, 50.000 cells per well were plated in 5.5 mM Glc, 2 mM Glut, 10% FBS in 6-well plates in duplicates. The next day, the medium was changed and cells were grown for 48 h. Before harvesting, cells were 90–100% confluent. Cells were washed 3 times with cold PBS, and scraped in 0.5 mL PBS. Two wells were pooled into one 1 mL sample and pelleted by centrifugation at 2000× *g*, 5 min, at 4 °C. The supernatant was then removed and the pellet frozen at −80 °C before analysis. Lipidomics analysis was performed at the Lipometrix facility at KU Leuven technology platform as previously described [29,30]. The data were represented as relative abundances (fold change, FC) compared to healthy controls.

### 2.9. Statistics

While the experiments were repeated and all the sample conditions were performed in duplicates or triplicates, statistical tests were not performed due to the lack of sufficient power (patient n = 1). Unless otherwise described, error bars were 95% Confidence Intervals.

## 3. Results

### 3.1. Clinical Presentation

The patient presented within the first six months of life with global developmental delay, dystrophy and a Leigh-like MRI pattern, as well as elevated lactate levels in blood, CSF and MRS, suggestive of Leigh syndrome (Table 1). Other clinical signs not commonly associated with Leigh syndrome, such as bifid uvula, were also present. Birth weight and length were <3 centile (2530 g, 46 cm). Parents were consanguineous of Turkish origin. Muscle biopsy was performed at age 1 year and showed atrophy of type II fibers, with no ragged-red fibers and normal a COX^-^/SDH^+^ ratio. Development was severely impaired and the patient developed early bilateral hearing loss. She also presented with truncal muscular hypotonia, and later developed a spastic dystonic movement disorder and epilepsy. A second muscle biopsy was performed at 11 years of age, showing reduced activities of respiratory chain complexes I–IV. Based on the patient’s clinical presentation, Leigh syndrome was suspected and Whole Exome Sequencing (WES) was performed at 14 years of age, revealing a homozygous, previously reported [8], pathogenic variant in *NDUFA13* (NM_015965.7(NDUFA13): *c.170G>A* (p.(Arg57His)). Surprisingly, WES also showed a homozygous (likely) pathogenic variant in *PGM1* (c.1108A>T, (p.(Lys370*)), which was previously not reported.

Based on genetic findings, testing for glycosylation abnormalities was performed revealing a mixed CDG I/CDGII type in serum consistent with PGM1-CDG [12,16,18]. Unfortunately, the patient passed away at 16 years of age due to respiratory failure caused by an influenza A infection with bacterial superinfection, requiring ventilation and tracheostomy. She could not be weaned from the respirator and after several episodes of lactic acidosis, developed intracranial pressure with blurred pupils. The patient died after deciding against further intensification of therapy due to increasing circulatory failure. Unfortunately, galactose treatment could not be initiated.

**Table 1:** Proband’s clinical characteristics and their overlap with PGM1-CDG or NDUFA13/Leigh syndrome patients.

	Proband	PGM1-CDG	Leigh Syndrome	*NDUFA13* Deficiency
Gene(s) affected	*PGM1, NDUF13A*	*PGM1*	>75 genes involved in OXPHOS and OXPHOS assembly, stability and activity. Most commonly associated with complex I deficiency (>1/3 of patients) [9,11,31].	*NDUFA13*
Genome	Nuclear	Nuclear	Nuclear, mitochondrial	Nuclear
Frequency	First case with combined PGM1 and NDUFA13 deficiency.	Not known. More than 70 patients reported to date [15,32].	1:40,000 [9,10], varies between different affected genes.	Not known, 13 patients 11 families reported [6,7,8].
Age of onset	Presented with bifid uvula at birth. In the first six months of life, presented with global developmental delay and dystrophy.	Mostly at birth [16], one adult case reported [20].	Variable, adult/adolescent onset reported but infrequent [9,31,33].	11/13 [8] patients presented within the first year of life.
Life expectancy	Died at the age of 16 years.	Oldest reported patient is 53 years [20] old; early death due to cardiac complications has been reported in 10% of the patients [16,21,22].	Depends on the genetic defect; in general, severely reduced [9,31], many patients die in infancy.	12/13 [6,7,8] patients still alive at the time of this publication. Average age 7.8 years (±5.4 years).
Prenatal/perinatal complications	Not reported.	Frequent [16], mostly associated with dysmorphic facial features resulting in breathing and feeding difficulties.	Infrequent [9]	Not reported.
Progression	Yes	Usually, stable.	Yes, fast, death at early age [9].	11/13 slow-moderate progression [6,7,8].
Congenital malformations	Bifid uvula	Bifid uvula, PRS and cleft palate [16].	Infrequent, varies between different affected genes [9].	Skeletal deformities and congenital glaucoma in one individual [8].
Growth delay	Severe	Frequent	About half of the patients [9].	9/13 patients [6,7,8].
Brain findings	Leigh like MRI pattern. Elevated lactate on MRS.	Majority unremarkable [16]. Thin pituitary reported in one patient [34].	Bilateral symmetrical lesions within the brainstem and basal ganglia (“Leigh like MRI pattern”) cerebellar atrophy, thalamus and spinal cord can also be affected, elevated lactate on MRS [9,31].	11/13 [6,7,8], progressive cerebellar atrophy with hypersignal of the cerebellum and dentate nucleus; atrophic optic nerves; elevated lactate on MRS.
Other neurological presentation	Dystonic movement disorder, epilepsy and ataxia.	Present in <40% of patients, mostly related to intellectual disability [19], seizures reported in only 3 patients [16,17,18,34].	Common [14], ataxia, abnormal spasticity and seizures.	13/13 [6,7,8] patients, seizures, intellectual disability, bradykinesia, spasticity, axial hypotonia and truncal ataxia.
Developmental delay	Severe	Often [16]	The majority of the patients [9].	13/13 [6,7,8], ranging from mild to severe.
Regression	Present	Infrequent [16]	Frequent [9]	Yes
Intellectual disability	Severe	Present in <40%, mostly due to complications of hyperinsulinemic hypoglycemia [19].	Frequent [9]	8/13 [6,7,8] patients, mild to severe.
Eye presentation	Absent	Infrequent, 5 reported patients [16], nystagmus and strabismus.	Varies between different affected genes [9], nystagmus and optic atrophy.	11/13 patients [8], nystagmus, optic atrophy and congenital glaucoma.
Hearing loss	Early bilateral hearing loss.	Infrequent, 1 reported patient [16,26].	Varies between different affected genes [9].	One patient with sensorineural loss [6,8].
Cardiovascular	Not reported.	Approximately half of the reported patients present with cardiac abnormalities [15,16,32].	Uncommon [9], in some patients ventricular septal defects and hypertrophic cardiomyopathy [35].	Reported in 1 patient, hypertrophic cardiomyopathy [7].
Musculoskeletal	Atrophy, truncal hypotonia.	Mare than 70% of the patients [16], rhabdomyolysis, hypotonia and elevated CK.	Frequent [9], including dystonia, hypotonia.	10/13 [6,7,8] including hypertonia, hypotonia, truncal hypotonia and decreased muscle tone.
Gastrointestinal	Not reported.	GI problems uncommon [16], mostly associated with feeding difficulties due to facial dysmorphism.	Varies between different affected genes [9], dysphagia, feeding difficulties and vomiting.	1/13 [6] Gastroesophageal reflux.
Liver	Mildly elevated liver transaminases.	Common [16], elevated liver enzymes, liver steatosis	Depends on the genetic defect, not frequently affected [9].	Not reported.
Endocrine	Elevated TSH.	Common [16], hypothyroidism, hypogonadotropic hypogonadism, delayed puberty and hyperinsulinemia.	Common [36], depending on the genetic defect.	Not reported.
Complex I activity	All complexes including CI were reduced in the muscle.	Not reduced in fibroblasts [18].	Depends on the genetic defect.	6/13 patients tested [6,7,8], isolated CI deficiency (muscle or fibroblasts).
Muscle biopsy	Muscle biopsy negative for ragged fibers, positive for atrophy of type II fibers.	Not usually performed, increase of internal nuclei or fiber size variation and/or accumulation of fat or glycogen was reported [12,16].	Frequently performed, muscle biopsy can show ragged fibers [33].	1 patient with showed lipid droplets in type I fibers with no ragged-red or cytochrome c oxidase negative fibers [7].
Biochemical laboratory findings	Elevated CSF and plasma lactate.	Common [16], low FSH, LH, IGFB and thyroid hormones; low glucose, coagulation factor abnormalities, elevated liver enzymes and elevated CK. Lactate not commonly elevated.	Elevated CSF and plasma lactate. Elevated amino acids. Elevated urinary organic acids. Abnormal plasma carnitine panel.	Elevated plasma lactate 7 patients, elevated plasma alanine 3 patients and elevated CSF lactate in 2 patients [8].
Serum Transferrin	Mixed CDG type.	Mixed CDG type, or in rare cases, CDG type II [16].	Not routinely performed.	Not performed.

### 3.2. Mitochondrial Functional Assays

**Figure 2:**
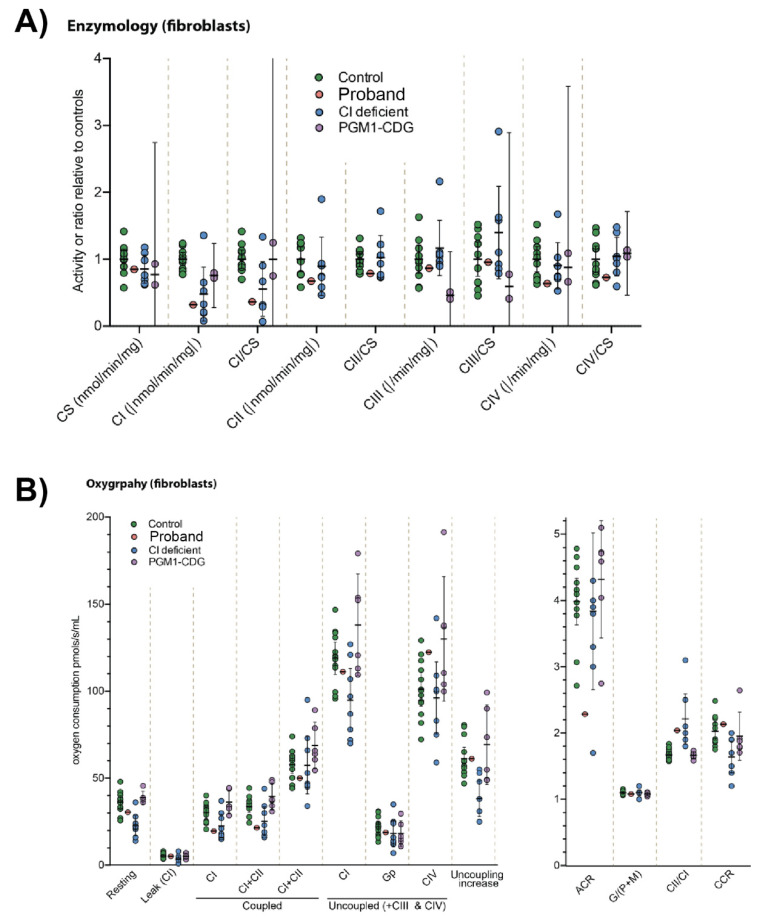
(**A**)**.** OXPHOS complexes enzymology results in control, CI-deficient, PGM1-deficient and proband fibroblast cell lines. Healthy control, OXPHOS CI (CI-deficient), PGM1-CDG and the proband’s fibroblasts were assessed for citrate synthase (CS) and respiratory chain complex I–IV (CI–IV) activity by spectrophotometry. Results are depicted as either raw activity rates or relative to CS activity. Median with error bars showing the 1.25th and 98.75th percentiles of the reference range are shown. Each data point represents the average of each patient or control from ≥2 technical replicates. Proband n = 1; CTR n = 9; PGM1-CDG n = 2; CI deficient n = 7. (**B**)**.** Oxygraphy results in control, CI-deficient, PGM1-deficient and proband fibroblast cell lines. Healthy control, OXPHOS CI (CI-deficient), PGM1-CDG and proband were subjected to oxygraphy and different OXPHOS complexes assessed. Resting, coupled and uncoupled rates of respiration are shown on the left, whereas ratios and calculated values from data in left panel are shown on the right. Median is displayed with error bars showing the 1.25th and 98.75th percentiles of the reference range. Each data point represents the average of each patient or control from ≥3 technical replicates. Proband n = 1; CTR n = 11; PGM1-CDG n = 6; CI deficient n = 7. Abbreviations: ACR—acceptor control ratio; CI–IV—respiratory chain complexes I–IV; CCR—coupling control ratio maximal uncoupled activity over maximal coupled activity; CS—citrate synthase G—glutamate; M—malate; and P—pyruvate.

Previously, we have thoroughly investigated CI-deficient [27,28] and PGM1-CDG fibroblasts [18] with the mitochondrial functional assays. To probe the oxidative phosphorylation (OXPHOS) in the fibroblasts of our patient, we performed complex enzyme measurements and respirometry (oxygraphy) on the proband’s fibroblasts and the fibroblasts of individuals with a known complex I (CI) or PGM1-deficiency (Supplementary Table S1). The enzymology studies showed the proband’s cells had a reduction in CI activity (Z-score = −4.5), comparable to the known CI-deficient fibroblasts. (Figure 2A). While CIV activity was reported as increased in other *NDFA13* individuals [8], CIV activity in our patient’s fibroblast was on the lower range of the CTR (Z-score = −1.4) (Figure 2A). When assessed by respirometry, the proband’s fibroblasts also showed an isolated CI deficiency profile with decreased CI activity (Z-score = −2) and increased CII/CI ratio (Z-score = 4.4), whereas the PGM1-CDG fibroblasts did not show any significant OXPHOS disturbance (Figure 2B).

Together, these results indicate the proband’s fibroblasts have an isolated CI deficiency, as seen in other CI defects.

### 3.3. Tracer Metabolomics in Proband’s Fibroblasts

To understand the metabolic consequences of the combined decreased CI and PGM1 activity, we next performed tracer metabolomics analysis on the proband’s fibroblasts.

**Figure 3:**
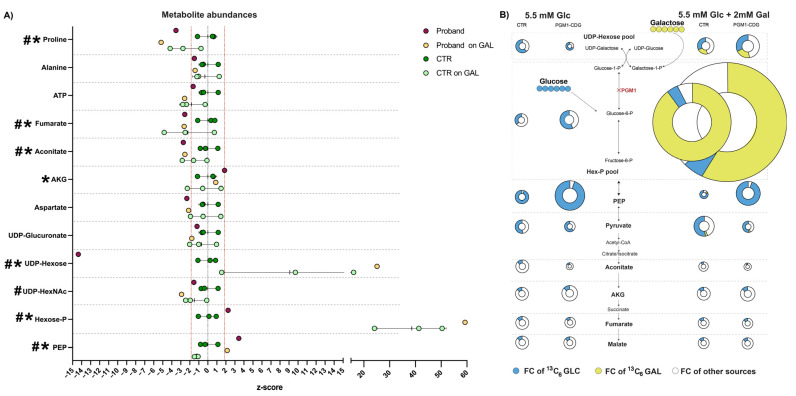
Metabolomics results in control and proband fibroblast cell lines with and without D-galactose treatment. (**A**) Intracellular metabolite abundances are represented as z-scores compared to healthy controls. Z-scores below −1.96 or above 1.96 are consistent with *p* < 0.05 in a two-tailed *t*-test. Metabolite abundances in the GLC-only condition with z-score below −1.96 and above 1.96 are marked with a *. Metabolite abundances in GLC+GAL condition with z-scores below −1.96 and above 1.96 are marked with a #. Z-score cut-off values are indicated with a red dashed line. (**B**) Fractional contribution (FC) of 5.5 mM ^13^C_6_-glucose (Glc) or ^13^C_6_-galactose (Gal) to different metabolites. The FC of ^13^C_6_-Glc is indicated by blue, while the FC of ^13^C_6_-Gal is indicated by yellow. The size of the pie indicates the relative abundance of the metabolite compared to the average of the control on 5.5 mM glucose conditions. The details are given in the Supplementary Materials. Proband n = 1, *t* = 2, CTR n = 3, *t* = 1–2. UDP-hexose pool- pool of UDP-glucose and UDP-galactose (these metabolites cannot be separated well by LC/MS method used), HexP pool—Hexose-Phosphate pool- pool of glucose-6-P, glucose-1-P, fructose-6-P, galactose-1-P, etc. (these metabolites cannot be separated well by LC/MS method used). Abbreviations: AKG—alphaketoglutarate; FC—fractional contribution; Glc—glucose; Gal—galactose; Hex-P—hexose-phosphate; and PEP—phosphoenolpyruvate.

Previously, similar tracer metabolomics experiments were performed on fibroblasts of patients with PGM1-CDG [18] and OXPHOS defects [28,37,38]. Therefore, to correctly interpret the results and compare them to previous findings, we calculated z-scores for different metabolites, where z-scores lower than −1.96 or higher than 1.96 were considered significant (*p* < 0.05, two-tailed test). We found that the top significantly changing metabolite in the proband was UDP-hexose (z-score −14.4) (Figure 3A), which is comparable to the results found in PGM1-CDG fibroblasts and consistent with PGM1 deficiency [18]. However, we found increased hexose-P (pool of galactose-1-P, glucose-1-P, mannose-1-P, mannose-6-p, fructose-6-p, etc.) in the proband compared to healthy controls (z-score 2.24). This is in contrast with our previously reported findings in PGM1-CDG fibroblasts, which showed a decrease in hexose-P (specifically galactose-1-P) [18]. Similarly, phosphoenolpyruvate (PEP) was increased in the proband compared to healthy controls (z-score 3.4). The increase in HexP and PEP suggests that glycolysis was increased in the proband’s fibroblasts. Other mitochondria-related metabolites such as aconitate (z-score −2.7), fumarate (z-score −2.6), aspartate (z-score −2.3) and proline (Pro, z score −3.5) were decreased in the proband. Alpha-ketoglutarate (oxoglutarate; AKG) was increased in the proband’s fibroblasts, however, not significantly (z-score 1.84). Those findings match previously described metabolic consequences of OXPHOS deficiency [28].

Previously, we have reported that 2 mM Gal supplementation resulted in the increase of UDP-hexoses and hexose-P in the fibroblasts of individuals with PGM1-CDG [18]. To investigate whether D-gal would be able to correct our patient’s phenotype, we also tested the effect of D-gal supplementation in our patient’s fibroblasts by incubating the fibroblasts with either (A) 5.5 mM ^13^C_6_-glucose, (B) 5.5 mM ^13^C_6_-glucose + 2 mM ^12^C_6_-galactose or (C) 5.5 mM ^12^C_6_-glucose + 2 mM ^13^C_6_-galactose. We found that Gal increased UDP-hexose and hexose-P in our patient’s fibroblasts (Figure 3A,B), and the majority of the increase was coming from Gal (Figure 3B). These results were comparable to our previous findings in PGM1-CDG fibroblasts [18]. However, the abundances of the TCA cycle metabolites—aconitate, fumarate, proline—which were decreased in the proband’s fibroblasts, did not improve in the presence of 2 mM Gal, nor did we observe any fractional contribution (FC) of ^13^C_6_-Gal to any of the TCA cycle metabolites (Figure 3B). Moreover, Gal further decreased ATP and aspartate (Figure 3A) and increased the abundance of arginine, ornithine and citrulline, metabolites involved in the urea cycle (Supplementary Figure S1).

### 3.4. Lipidomics in Fibroblasts

**Figure 4:**
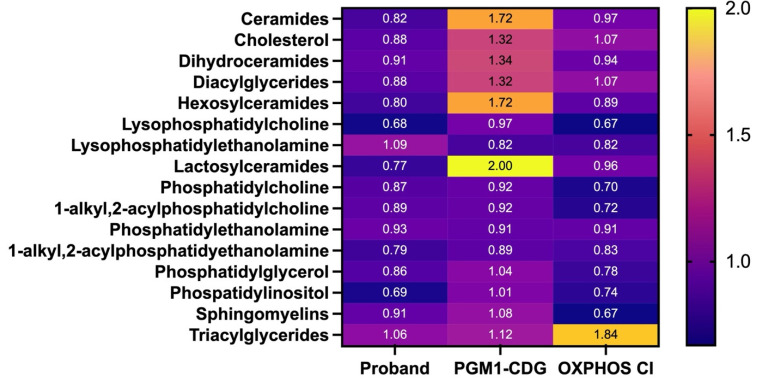
Heatmap showing lipidomics results. Proband, PGM1-CDG (n = 2, *t* = 2) and CI-deficient (n = 2, *t* = 2) fibroblasts were collected, and their lipidomics composition compared to the healthy controls (n = 2, *t* = 2). The fold change (FC) of the lipids compared to healthy controls is indicated by the color, where darker color indicates lower abundance and lighter color (yellow) higher abundance.

To further investigate the metabolic consequence of the PGM1 and NDUFA13 deficiency, we performed lipidomics experiments. To this end, we compared fibroblasts from the proband, PGM1-CDG individuals and OXPHOS CI-deficient individuals. We found that the lipidomic profile of the proband showed prominent differences compared to healthy controls with both similarities and differences with PGM1-CDG and CI-deficient fibroblasts (Figure 4). In proband fibroblasts, compared to controls, most prominent differences were found in lysophosphatidylcholine (LPC) and phosphatidylinositol (PI) (average FC = 0.67; 0.68, respectively), with a similar trend in CI-deficient cells, but not in PGM1-CDG. On the other hand, triacylglycerides (TAGs) were more increased in CI-deficient fibroblasts (average FC = 1.84) compared to proband and PGM1-CDG fibroblasts (Figure 4). Sphingomyelins were most decreased in OXPHOS CI cells (average FC = 0.67). Strikingly, lactosylceramides (average FC = 2), ceramides (average FC = 1.72), hexosylceramides (average FC = 1.72), dihydroceramides (average FC = 1.34) and cholesterol (average FC = 1.32) were increased in PGM1-CDG fibroblasts, while the levels of these lipids in the proband and the CI-deficient cells were similar to the controls. These results suggest that the fibroblasts of our patient show overlap with both PGM1-CDG and CI-deficient fibroblasts, with most prominent changes in LPC and PI.

## 4. Discussion

Here, we present the first case with dual diagnosis of CDG and Leigh syndrome caused by (likely) pathogenic variants in *PGM1* and *NDUFA13* (Figure 1). On the one hand, the clinical presentation consistent with NDUFA13 deficiency in our patient included global developmental delay, seizures, dystrophy, hearing loss, a Leigh-like MRI pattern and high lactate levels in blood and CSF. On the other hand, PGM1-CDG like-presentation included bifid uvula, elevated TSH and abnormal glycosylation in blood. In addition, our patient had no eye abnormalities, which were reported in 11/13 of NDUFA13 patients [8], but are less common in PGM1-CDG [16].

While the homozygous c.1108A>T *PGM1* variant has not been reported so far, *NDUFA13* variant c.170GA>3 was previously reported in six families, in either a heterozygous or homozygous state [8]. The patients homozygous for the c.170G>A variant presented with cognitive impairment and tended to have slower disease progression compared to individuals with other pathogenic variants. They were still alive at the time of the publication, with their ages ranging from 2.6 to 18 years [8]. Unfortunately, our patient passed away at 16 years of age, which might suggest a more severe clinical presentation compared to the reported NDUFA13 individuals with the same homozygous variant. There are several reports of pediatric death in PGM1-CDG, and the leading cause of death in these patients was cardiac arrest [16]. However, our patient did not show cardiac involvement. Given the patient’s mixed clinical and biochemical presentation, as well as early death, we hypothesize both *NDUFA13* and *PGM1* variants were disease causing.

To confirm the NDUFA13 and PGM1 deficiency and probe the extent of their biochemical consequences in our patient, we turned to metabolic profiling tools such as oxygraphy and respiratory complex enzyme measurements by spectrophotometry, tracer metabolomics and lipidomics in the patient’s fibroblasts. Unfortunately, no other material was available due to the patient’s passing.

First, we assessed the effect of the complex I deficiency. The fibroblasts of previously reported NDUFA13 patients showed decreased CI activity and increased CIV activity [8]. Therefore, we expected to see similar effects in our patient’s fibroblasts. As we did not have any other NDUFA13-deficient patients in our center, we used the fibroblasts of other CI-deficient patients instead (Supplementary Table S1). In contrast to other CI-deficient cells, we only observed decreased CI activity (Figure 2A) in our patient’s fibroblasts. Unlike in other reported NDUFA13 patients, complex IV was also not increased in our patient’s fibroblasts. Similarly, PGM1-deficient fibroblasts did not have a reduced CI activity (Figure 2B).

Therefore, to evaluate possible metabolic re-wiring resulting from the dual deficiency in our patient’s fibroblasts, we performed tracer metabolomics experiments (Figure 3). We found UDP-hexose was significantly depleted, biochemically confirming the PGM1 deficiency in our patient. Similarly, aspartate was depleted in our patient’s fibroblasts, which was also previously reported in fibroblasts of patients with OXPHOS defects [28,37,38]. In addition, OXPHOS-deficient fibroblasts were shown to have increased upper glycolytic intermediates [28], which we also observed in our proband’s cells (Figure 3A).

However, we found alterations in several other metabolites, which were not reported in PGM1-CDG or OXPHOS defects. For example, though pyruvate was decreased in OXPHOS defects [28], it was not significantly different in our patient’s fibroblasts compared to the controls (Figure 3A). Further, while proline was significantly decreased in our proband’s fibroblasts (Figure 3A), it was significantly increased in patients with OXPHOS defects [28]. Nevertheless, we did observe a mild elevation in AKG in our proband’s fibroblasts (Figure 3A), comparable to the AKG increase reported in other OXPHOS defects [28]. Considering complex I provides NAD+ for the reaction needed to make succinyl-CoA from AKG (Figure 1) [39], the elevation of AKG is supportive of the decreased CI activity in our patient’s fibroblasts.

Though the abundance of several TCA cycle metabolites was affected in our proband’s fibroblasts compared to the controls (Figure 3A), we did not observe differences in the fractional contribution (FC) of glucose in the TCA cycle metabolites compared to the controls, as previously observed in OXPHOS defects [28]. On the other hand, the FC of ^13^C_6_-glucose to UDP-hexoses was decreased (Figure 3B), as expected in a PGM1 defect [18]. These findings indicate that the glucose is redirected away from glycosylation and glycogen metabolism, towards other pathways such as TCA cycle. Therefore, decreased PGM1 activity might ameliorate mitochondrial disturbances caused by CI deficiency.

Finally, when we treated our proband’s cells with Gal, we found a significant increase in UDP-hexoses and hexose-P, coming from Gal (Figure 3B). The increase in UDP-hexose is comparable to the one seen in PGM1-CDG fibroblasts [18], suggesting Gal treatment in the patient might have resulted in improved glycosylation. However, except for an increase in UDP-hexose (Figure 3), Gal treatment did not result in an improvement in other metabolites. Instead, Gal decreased ATP and aspartate in the proband’s cells and increased the abundance of arginine, ornithine and citrulline, metabolites involved in the urea cycle (Figure 3A, Supplementary Figure S1), indicating mitochondrial homeostasis was negatively impacted by the presence of Gal. In light of this, treatment with aspartate [37] has previously been shown not be beneficial for rescuing mitochondrial defects. However, it would be interesting to investigate the combined treatment of pyruvate and uridine, which was previously shown to improve the metabolome and respiratory capacity in CI-deficient fibroblasts [28], on the patient’s fibroblasts, though this is beyond the scope of this publication.

Lipids are a downstream product of acetyl-CoA and a major component of cell membranes, including mitochondria [40]. As lipids are closely related to mitochondrial function, and the changes in lipidome of both CDG [41] and mitochondrial disease patients [42] have already previously been reported, we pursued lipidomics for our patient’s fibroblasts. Strikingly, we found several lipid species to be altered in PGM1-CDG fibroblasts such as ceramides, hexosylceramides and lactosylceramides, which was not the case of our proband’s fibroblasts or CI-deficient fibroblasts (Figure 4). On the other hand, phosphatidylinositol (PI) and lysophosphatydilcholine were decreased in our proband’s fibroblasts, as well as in CI-deficient cells. Interestingly, sphingomyelin was affected in CI-deficient cells, and not in NDUFA13 cells (Figure 4). Sphingomyelin, and other lipids, are especially abundant in neurons, where they perform structural and supportive role [43]. As nervous involvement is usually present in Leigh syndrome [9], but not in PGM1-CDG [16], one could speculate whether the differences in the lipid content in these two disorders could account for such different neurologic presentations. Nevertheless, our proband’s fibroblasts showed milder changes in the lipidome profile compared to both PGM1 and CI-deficient cells, indicating that the two deficiencies might have had opposing effects on the patient’s lipidome.

In conclusion, our results support the dual diagnosis of PGM1-CDG and Leigh syndrome caused by NDUFA13 deficiency.

Further, our findings suggest that the consequences of PGM1 deficiency, such as decreased flux through glycosylation and glycogen metabolism, might partially attenuate the biochemical consequences of CI deficiency. Finally, apart from confirming the patient’s diagnosis, the insights generated in this study could be critical for mapping disease mechanisms and treatment options in PGM1-CDG and Leighs syndrome patients.

## Data Availability

All data generated in this study can be requested from the corresponding author (Eva Morava). Any additional information needed to analyze the data can be received from the lead contact upon request.

## Supplementary Materials

The following supporting information can be downloaded at https://www.mdpi.com/article/10.3390/cells14090638/s1, Figure S1: Amino acid abundances results control and proband fibroblast cell lines with and without D-galactose treatment. Intracellular metabolite abundances are represented as z-scores compared to healthy controls. Z-scores below −1.96 or above 1.96 are consistent with *p* < 0.05 in a two-tailed *t*-test. Z-cut off values are indicated in red dashed line; Table S1: Demographic and molecular characteristics of patients whose fibroblasts were used for the oxygraphy, enzymology, lipidomics and/or tracer metabolomics. Abbreviations: CDG—congenital disorder of glycosylation, CI—complex I, F—female, M—male, OXPHOS—oxidative phosphorylation and yo—years old. * Age reported in the first publication.
