# Supplementary material for: Complex Metabolomic Changes in a Combined Defect of Glycosylation and Oxidative Phosphorylation in a Patient with Pathogenic Variants in PGM1 and NDUFA13
Source: Cells. 2025 Apr 25;14(9):638. doi: 10.3390/cells14090638 (PMC12071635; doi:10.3390/cells14090638)
Supplement: Supplementary file 1 [file cells-14-00638-s001.zip › cells-3567913-supplementary.pdf]

Supplementary Materials:

Supplementary Table S1. Demographic and molecular characteristics of patients whose fibroblasts were used for the oxygraphy, enzymology, lipidomics and/or tracer metabolomics. Abbreviations: CDG—congenital disorder of glycosylation, CI—complex I, F—female, M—male, OXPHOS—oxidative phosphorylation and yo—years old. * Age reported in the first publication.

Identifier	Diagnosis	Age/Gender	Affected gene	Pathogenic variant
Proband	PGM1-CDG<br>Leigh Syndrome	16 yo*/F	*NDUFA13*<br>*PGM1*	*NDUFA13* c.170G>A, (p.57R>H);<br>*PGM1* c.1108A>T, (p.370K*)
P1	PGM1-CDG	16 yo*/F	*PGM1*	c.1508G>A homozygous
P2	PGM1-CDG	11 yo*/M	*PGM1*	*PGM1* c.787G>T, c.1551C>A<br>p.D263Y, p.Y517X
P3	PGM1-CDG	10 yo*/M	*PGM1*	*PGM1* c.1162G>A, c.1547T>C<br>p.E388K, p. L516P
P4	PGM1-CDG	11 yo*/M	*PGM1*	*PGM1* c.871G>A, c.1144+3A>T<br>p.G291R
P5	PGM1-CDG	20 yo*/F	*PGM1*	*PGM1* c.1145-222G>T, p.G382Vfs*2 homozygous
P6	PGM1-CDG	3 yo*/F	*PGM1*	*PGM1* c.551delT, p.F184Sfs*9, homozygous
P7	Leigh syndrome (OXPHOS CI)	Disease onset 2yo / deceased at 4 yo/M	*NDUFS1*	c.1057G>C, (p.353A>P), c.420+2T>C (splice site variant)
P8	Leigh syndrome (OXPHOS CI)	Diseased at birth/deceased before 1yo / F	*NDUFS2*	c.1336G>A, (p.446D>N)
P9	Leigh syndrome (OXPHOS CI)	Not known	*AIFM1*	
P10	Hypertrophic cardiomyopathy, lactic acidosis (OXPHOS CI)	Disease onset 3yo / survival >14 yo/ F	*ACAD9*	c.1687C>G, p.(H563D), c.380G>A p.(R127G)
P11	Hypertrophic cardiomyopathy, lactic acidosis (OXPHOS CI)	F	*ACAD9*	c.976G>C (p.A326T) and c.1552C>T (p.R518C)
P12	Leigh syndrome (OXPHOS CI)	Diseased at birth / deceased at 2yo / F	*MT-ND1*	mtDNA.3481G>A (p.59E>K) with 50% heteroplasmy
P13	Leigh syndrome (OXPHOS CI)	Disease onset 19yo / survival > 43yo / M	*MT-ND6*	mtDNA.14487T>C (p.63E*) with 5% heteroplasmy

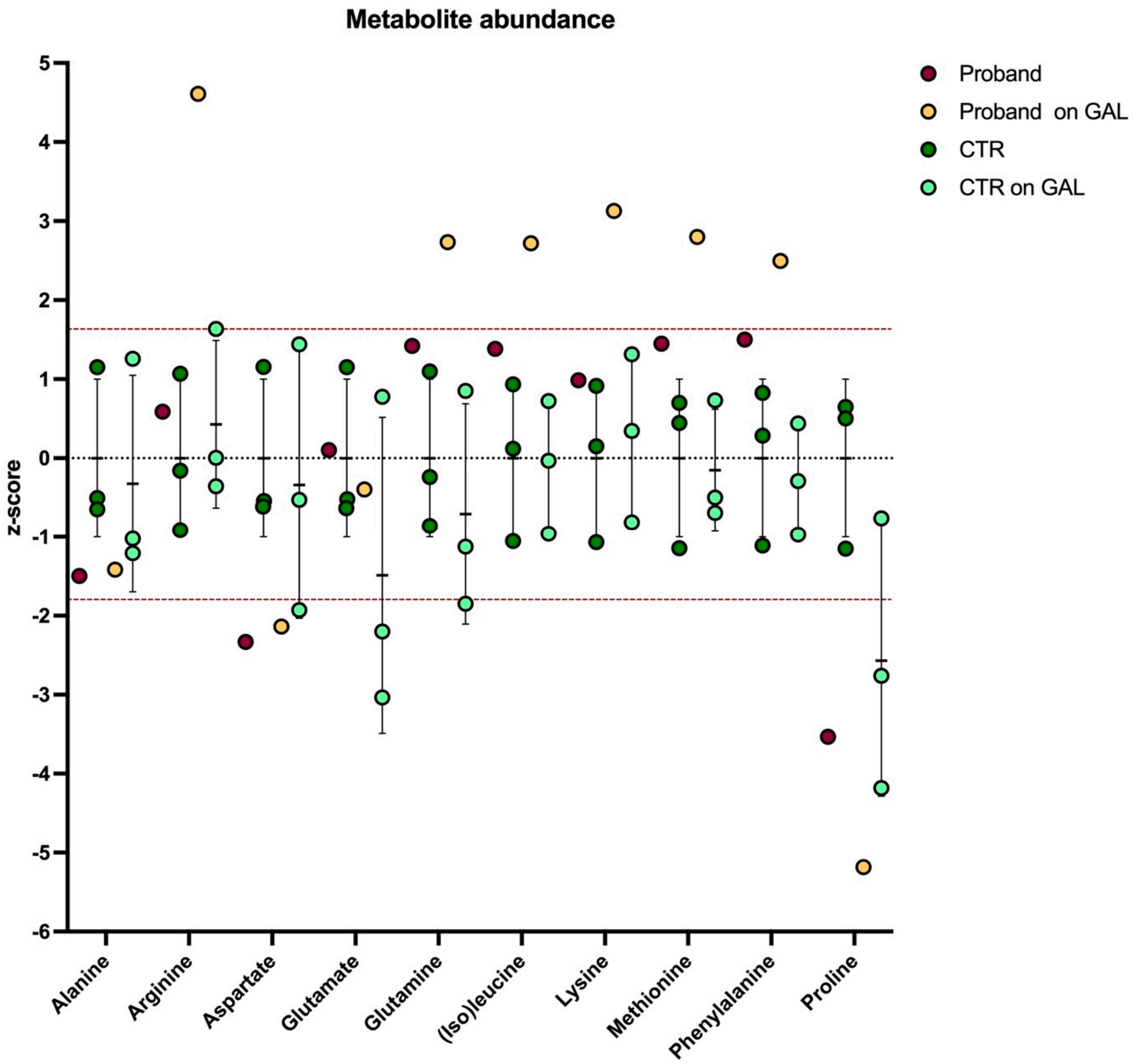

Supplementary Figure S1. Amino acid abundances results control and proband fibroblast cell lines with and without D-galactose treatment. Intracellular metabolite abundances are represented as z-scores compared to healthy controls. Z-scores below −1.96 or above 1.96 are consistent with $p < 0.05$ in a two-tailed *t*-test. Z-cut off values are indicated in red dashed line.
